# Recurrent hyperammonemic encephalopathy in a breast cancer patient receiving capecitabine therapy: a case report and literature review

**DOI:** 10.3389/fphar.2025.1715914

**Published:** 2026-01-20

**Authors:** Yuling Mu, Qing Li, Jilin Kong, Yuyao Zhao

**Affiliations:** 1 Department of Oncology, Yantaishan Hospital Affiliated to Binzhou Medical University, Yantai, Shandong, China; 2 Department of Breast Surgery, Yantaishan Hospital Affiliated to Binzhou Medical University, Yantai, Shandong, China

**Keywords:** 5-fluorouracil, breast cancer, capecitabine, hyperammonemic encephalopathy, S-1

## Abstract

Capecitabine-induced hyperammonemic encephalopathy represents a rare adverse event that can complicate clinical diagnosis. To accurately diagnose this condition, clinicians should consider a combination of factors, including the patient’s history of capecitabine use, clinical manifestations of altered mental status accompanied by hyperammonemia, and the exclusion of alternative causes for the altered level of consciousness. We report a case of a female patient with breast cancer who developed hyperammonemic encephalopathy during her first cycle of a capecitabine-containing chemotherapy regimen, with a peak plasma ammonia level of 191.4 μmol/L. Discontinuation of capecitabine, together with hemoperfusion, and other ammonia-lowering therapies, led to the normalization of ammonia levels, restoration of consciousness, and recovery of laboratory parameters and vital signs. The patient was subsequently switched to S-1, which she tolerated well for 14 months without significant adverse effects. However, upon reintroduction of a capecitabine-containing regimen due to disease progression, hyperammonemic encephalopathy recurred, with a peak ammonia level of 333.6 μmol/L. After cessation of capecitabine and initiation of plasmapheresis and other ammonia-reducing treatments, her ammonia levels normalized, consciousness was restored, and all laboratory and clinical parameters returned to normal. This case underscores the necessity of maintaining a high index of suspicion for rare adverse reactions, even in patients with complex medical histories. While it is typically more pragmatic to prioritize common etiologies initially, clinicians must remain open-minded in their differential diagnosis when conventional explanations fail to account for the clinical presentation.

## 1 Introduction

5-Fluorouracil (5-FU), a pyrimidine antimetabolite, has been a fundamental component in the systemic therapy of solid tumors, particularly those of the gastrointestinal, breast, and genitourinary systems, due to its effective inhibition of DNA synthesis in rapidly proliferating cells (Ciaffaglione et al., 2021). Capecitabine, a prodrug of 5-FU, undergoes enzymatic conversion to 5-FU in the plasma. Following oral administration, capecitabine is rapidly absorbed through the gastrointestinal tract. The majority of the prodrug is then hydrolyzed by hepatic carboxylesterase (CES) to form 5′-deoxy-5-fluorocytidine (5′-DFCR). Subsequently, 5′-DFCR is converted to 5′-deoxy-5-fluorouridine (5′-DFUR) by cytidine deaminase (CDA), an enzyme widely distributed in various tissues, including the liver, plasma, and tumor sites. Finally, 5′-DFUR is hydrolyzed by thymidine phosphorylase (TP) to yield the active metabolite, 5-fluorouracil (5-FU) (Aldeen Majeed et al., 2023). Its cytotoxic effect is more pronounced in tumor tissue compared to blood and other tissues, thereby enhancing targeted drug delivery and promoting selective activation. Capecitabine demonstrates favorable tolerance and reduced systemic toxicity relative to intravenous 5-FU therapy, leading to its widespread clinical application. The hybrid regimen of intravenous 5-FU, comprising an initial bolus followed by a continuous infusion, may confer a potential efficacy advantage in certain clinical contexts. However, this benefit is achieved at the expense of a distinct toxicity profile, notably a higher incidence of myelosuppression, and significantly compromised administration convenience. In contrast, capecitabine, administered orally, provides comparable therapeutic efficacy to the intravenous 5-FU regimen while substantially improving treatment convenience and patient-reported quality of life. This advantage is particularly pronounced in elderly populations (Alzahrani et al., 2023).

S-1 is another oral fluoropyrimidine formulation composed of three distinct agents: tegafur (FT), a prodrug metabolized to 5-FU primarily by hepatic CYP2A6; gimeracil (CDHP), which inhibits dihydropyrimidine dehydrogenase to prevent 5-FU degradation; and oteracil (OXO), which reduces gastrointestinal toxicity by inhibiting orotate phosphoribosyltransferase in the gut (Kobayakawa and Kojima, 2011). Extensive clinical evidence from Asian populations supports the non-inferior efficacy and acceptable safety profile of S-1 relative to intravenous 5-FU or capecitabine. In Western populations, although data are more limited, findings from the SALTO trial indicate a favorable tolerability profile for S-1, including a reduced incidence of hand-foot syndrome (HFS), positioning it as a viable alternative for patients intolerant to 5-FU or capecitabine (Derksen et al., 2022). It is noteworthy that CYP2A6 activity demonstrates notable interethnic variation, with Caucasian populations exhibiting a higher prevalence of high-activity alleles, thereby resulting in increased systemic exposure to 5-FU. Accordingly, dose reduction of S-1 is advised in Western patients to mitigate potential toxicity (Kobayakawa and Kojima, 2011).

Despite their established roles in oncology, 5-FU and its prodrugs are associated with a spectrum of adverse effects. The toxicities of 5-FU and its prodrugs (capecitabine, S-1) primarily result from their effects on rapidly proliferating cells, with common adverse events including myelosuppression, gastrointestinal reactions (e.g., diarrhea, stomatitis), and hand-foot syndrome. Specifically, 5-FU frequently induces neutropenia. Capecitabine is associated with a higher incidence of hand-foot syndrome, attributed to its tissue distribution profile (Singh et al., 2017). In contrast, S-1, formulated with gimeracil and oteracil potassium, demonstrates reduced myelosuppression and diarrhea, though nausea and anorexia are more frequently observed (Saif et al., 2009). A notable yet infrequent adverse effect associated with these medications is hyperammonemic encephalopathy, which is characterized by elevated ammonia levels and altered mental status in the absence of radiologic abnormalities. The overall incidence of hyperammonemic encephalopathy with various fluoropyrimidine drugs has been reported at approximately 0.6% (Mitani et al., 2017). Although this condition is rare, it presents a significant risk to patients undergoing chemotherapy, necessitating prompt identification and intervention to avert severe outcomes (Jin et al., 2025). In this report, we present a case of hyperammonemic encephalopathy in a patient with advanced breast cancer who was treated with capecitabine. Additionally, we provide a review of the literature and emphasize the importance of recognizing and addressing precipitating factors that may predispose patients to the development of hyperammonemic encephalopathy when treated with capecitabine.

## 2 Case description

### 2.1 Patient history and initial presentation (2021)

The patient, a 68-year-old female, had previously undergone a right breast modified radical mastectomy at an external facility on 14 October 2016. Postoperative pathological analysis identified invasive lobular carcinoma within the breast tissue. The tumor measured 2.5 × 0.6 cm, with the nipple remaining free of tumor involvement; however, metastasis was detected in seven out of eight axillary lymph nodes. The postoperative pathological stage is determined as pT2N2M0, Stage ⅢA. Immunohistochemical findings indicated that the ER (3+), PR (−), HER-2 (3+), EGFR (2+), and P53 (−), along with an approximate 30% Ki67 expression. The molecular subtype was identified as HER2-positive, HR-positive breast cancer. The patient received adjuvant chemotherapy consisting of four cycles of EC (epirubicin, cyclophosphamide) followed by four cycles of T (liposomal paclitaxel). Although the historical IHC report from 2016 confirmed HER-2 positivity (3+), the patient did not receive any anti-HER-2 therapy after surgery. Subsequent to chemotherapy, the patient underwent radiotherapy and completed a 5-year course of anastrozole, concluding in January 2021. On 26 February 2021, the patient was admitted to the Department of Breast Surgery at our institution, presenting with a 1-month history of swollen supraclavicular lymph nodes. The patient had no documented history of hepatitis, liver cirrhosis, or cerebrovascular diseases. Baseline routine blood and chemistry analyzes revealed no significant abnormalities. Notably, liver function tests were within normal limits (ALB: 42.5 g/L, ALT: 11.4 U/L, AST: 15 U/L, TBIL: 11.3 μmol/L). Blood ammonia concentration is not typically included in routine laboratory assessments; consequently, it was not measured during the initial clinical evaluation. Non-contrast MRI of the brain revealed ischemia in the right basal ganglia and centrum semiovale. Whole-body MRI with diffusion-weighted imaging (WB-DWI/MRI) demonstrated widespread lymph node metastases, including the cervical, supraclavicular, infraclavicular, axillary, mediastinal, retroperitoneal, pelvic cavity, and inguinal areas, with no evidence of metastatic disease elsewhere. Fine-needle aspiration biopsy of the supraclavicular lymph node indicated the presence of metastatic carcinoma, which was clinically and pathologically identified as originating from the breast. Immunohistochemical analysis revealed estrogen receptor (ER) positivity, progesterone receptor (PR) negativity, HER-2 expression at a level of 2+, and a Ki-67 proliferative index of 30%. The dual-color silver *in situ* hybridization (DSISH) test was negative for HER-2 gene amplification. At the time of admission, the patient was assessed with a performance status (PS) score of 1 and was free of neurological symptoms, deeming them well-suited to proceed with chemotherapy. Postoperative pathology confirmed a diagnosis of HER-2 positive breast cancer. The patient did not receive targeted anti-HER-2 therapy during adjuvant treatment; Therefore, trastuzumab was initiated as part of the first-line therapy. Systemic chemotherapy commenced with a regimen of TXH, consisting of trastuzumab at 8 mg/kg on day 1, nab-paclitaxel at 260 mg/m^2^ on day 1, and capecitabine at 1,250 mg/m^2^ twice daily for 14 days, beginning on 6 March 2021, for the first two cycles.

### 2.2 First episode of hyperammonemic encephalopathy

#### 2.2.1 Diagnosis and management

Five days following the administration of capecitabine in the first cycle, the patient experienced a collapse in the bathroom. Upon examination, the patient’s Glasgow Coma Scale score was recorded as 11 (E3, V3, M5). According to standard clinical classification, a GCS score of 9–12 is indicative of a moderate impairment of consciousness. There were no accompanying symptoms such as vomiting, convulsions, or urinary or fecal incontinence. Electrocardiogram monitoring revealed a blood pressure of 125/79 mmHg, a heart rate of 78 beats per minute, and an oxygen saturation level of 100%. Physical examination indicated a body temperature of 36.5 °C, pupil diameters of approximately 3 mm, bilaterally equal and round pupils, and sensitivity to light reflex. No abnormalities were detected in cardiac and pulmonary auscultation, and there was an absence of neck stiffness, with normal muscle strength and tone in the limbs. However, bilateral positive Babinski signs were observed. Arterial blood gas analysis demonstrated metabolic acidosis with respiratory alkalosis. Hematological analysis revealed a significant elevation in blood ammonia levels, recorded at 199.80 μmol/L, compared to the normal reference range of 11–32 μmol/L. Liver function tests indicated mildly elevated enzyme levels, including ALT at 64.2 U/L, AST at 42.4 U/L, TBIL at 37.49 μmol/L, DBIL at 8.51 μmol/L, and IBIL at 29.0 μmol/L. PLT was reduced to 54 × 10^^9^/L (Table 1). An emergency CT scan of the brain appeared normal, while an emergency MRI revealed stenosis of the A1 terminal segment in the left anterior cerebral artery, as well as ischemia in the right basal ganglia and centrum semiovale. Upon the diagnosis of unexplained hyperammonemia, the patient was promptly admitted to the intensive care unit (ICU). The therapeutic protocol implemented in the ICU comprised hemodiafiltration with hemoperfusion, intravenous administration of branched-chain amino acids, and the administration of a lactulose enema to mitigate elevated blood ammonia levels (Table 2).

**TABLE 1:** Comparison of clinical and laboratory characteristics between the two hyperammonemic episodes.

Date	Symptoms/Signs	Peak ammonia level	Key lab abnormalities	Interventions to reduce blood ammonia levels	Outcome
2021/3	moderate consciousness impairment; pupils approximately 3 mm, sensitivity to light reflex, bilateral positive Babinski signs, and no other obvious abnormal signs	199.80 µmol/L	pH 7.39 pO_2_ 187.20 mmHg pCO2 25.4 mmHg SBE -9.2 mmol/L Lactic acid 2.7 mmol/L ALT 64.2 U/L AST 42.4 U/L Urea 10.87 mmol/L Crea 78.4 µmol/L	Hemodiafiltration with hemoperfusion; Intravenous infusion of branched-chain amino acids; Lactulose enema	recovery
2023/1	severe consciousness impairment; pupils approximately 5 mm, a sluggish pupillary light reflex, neck stiffness, bilateral lower limb rigidity, bilateral positive babinski signs	333.60 µmol/L	pH 7.2 pO_2_ 148.0 mmHg pCO_2_ 17.8 mmHg SBE -21.1 mmol/L Lactic acid 10.3 mmol/L ALT 180.70 U/L AST 227.20 U/L Urea 8.39 mmol/L Crea 84.60 mmol/L	Plasma exchange; Intravenous infusion of branched-chain amino acids; Lactulose enema	recovery

**TABLE 2:** Comparison of detailed laboratory parameters between the two episodes of capecitabine-induced hyperammonemic encephalopathy.

Laboratory parameter	Reference range	First episode of hyperammonemic encephalopathy	Second episode of hyperammonemic encephalopathy
PH	7.35-7.45	7.39	7.20
pO2	83-108 mmHg	187.2	148.0
pCO2	35-45 mmHg	25.4	17.8
AB	21.4-27.3 mmol/L	14.9	7.0
SBE	-3.0-3.0 mmol/L	-9.2	-21.1
Lac	0.5-1.6 mmol/L	2.7	10.3
Ammonia	11-32 µmol/L	199.8	333.6
AST	13-35 U/L	42.4	227.2
ALT	7-40 U/L	64.2	180.7
TP	65-85 g/L	65.8	64.1
ALB	40-55 g/L	39.9	39.1
TBIL	0.1-21 µmol/L	37.49	18.31
DBIL	0.1-3.4 µmol/L	8.51	5.94
IBIL	2-20 µmol/L	29.0	12.37
ALP	50-135 U/L	72.5	81.2
Urea	3.1-8.8 mmol/L	10.07	8.39
Crea	41-81 µmol/L	78.4	84.64
Na	137-147 mmol/L	138.2	134.0
K	3.5-5.3 mmol/L	4.01	4.43
CL	99-110 mmol/L	105.3	105.4
Ca	2.11-2.52 mmol/L	2.01	0.45
GLU	3.9-6.1 mmol/L	6.57	7.65
WBC	3.5-9.5×10^^9^/L	4.79	3.37
PLT	125-350×10^^9^/L	54.0	214.0
Hg	115-150 g/L	134.0	122.0

pO2, arterial partial pressure of oxygen; pCO2, arterial partial pressure of carbon dioxide; AB, actual bicarbonate; SBE, standard base excess; Lac, Lactic acid; AST, aspartate aminotransferase; ALT, including alanine aminotransferase; TP, Total protein; ALB, serum albumin; TBIL, Total bilirubin; DBIL, Direct bilirubin; IBIL, Indirect bilirubin; ALP, Alkaline phosphatase; Urea, Urea nitrogen; Crea, Creatinine; Na, serum sodium; K, serum potassium; CL, serum chloride; Ca, serum calcium; GLU, Glucose; WBC, white blood cell count; PLT, Platelet count; Hg, Hemoglobin.

#### 2.2.2 Outcome

**FIGURE 1:**
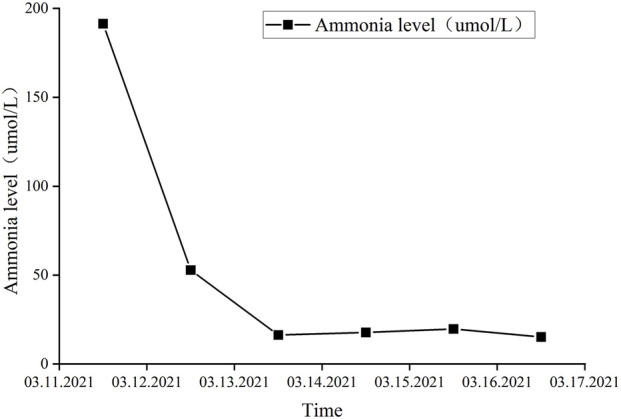
Blood ammonia levels in 2021.

After a 5-day course of treatment in the ICU, the patient regained consciousness, and laboratory evaluations demonstrated significant normalization, with blood ammonia levels measuring 17.7 μmol/L (Figure 1) and lactate levels at 0.9 mmol/L. Subsequently, the patient was transferred to the general ward and was discharged following notable clinical improvement.

### 2.3 Interim treatment and second presentation (2023)

Subsequently, the patient received chemotherapy regimens including trastuzumab, fulvestrant, S-1, and vinorelbine. Despite the patient demonstrating good tolerance to these treatments, there was no sustained therapeutic response. Throughout the treatment course, evaluations were conducted in a timely manner according to the Response Evaluation Criteria in Solid Tumors, version 1.1 (RECIST 1.1). The patient received first-line therapy with trastuzumab and fulvestrant. Surveillance imaging during treatment demonstrated slow progression in some metastatic lymph nodes. After 5 months, a follow-up scan revealed a new soft tissue mass medial to the left kidney, suggestive of metastasis. Based on these radiologic findings, the patient’s disease was comprehensively assessed as progressive disease (PD). The patient received a second-line regimen consisting of continued trastuzumab (for a total course of 1 year) combined with the oral chemotherapeutic agent S-1. During this period, follow-up assessments demonstrated significant reduction of some metastatic lymph nodes, while the remaining lesions remained stable. This second-line therapy was maintained for 14 months. Subsequently, a follow-up imaging scan in November 2022 confirmed disease progression (PD), characterized by significant enlargement of the left renal medial soft tissue mass and a newly detected punctate enhancing focus in the right insular lobe, which was suspicious for metastasis. Following the refusal of a repeat biopsy by the patient and family, vinorelbine monotherapy was administered in accordance with the 2022 NCCN and CSCO guidelines and the family’s expressed preferences. The follow-up scan after two cycles revealed disease PD, characterized by continued growth of the left renal medial lesion and new punctate enhancing foci in the right insular lobe and cerebellum, thereby necessitating a modification of the treatment plan.

Accordingly, a multidisciplinary (MDT) discussion was held regarding subsequent therapy. The patient’s treatment history following recurrence showed cautiously administered regimens with no significant adverse events but limited clinical benefit. Due to rapid progression over 2 months, a shift in strategy to more intensive combination chemotherapy was recommended. Although paclitaxel plays a critical role in breast cancer chemotherapy, it was not systematically administered following postoperative recurrence and metastasis of this patient. Additionally, liposomal paclitaxel had been previously utilized as an adjuvant chemotherapy for four cycles, demonstrating good tolerance without significant adverse events. Therefore, its application is recommended. The selection of combination therapy was based on the patient’s previous favorable clinical benefit from S-1, which was administered for over 1 year. Capecitabine, as another prodrug of 5-FU, was therefore reintroduced. During the 2021 presentation of hyperammonemic encephalopathy, concurrent medications included trastuzumab, nab-paclitaxel, and capecitabine. The etiology could not be conclusively ascribed to which one of these medications. Furthermore, the patient had previously tolerated S-1 well without experiencing any serious adverse events. Consequently, a causal association between hyperammonemic encephalopathy and capecitabine was not initially established. Upon comprehensive counseling, the patient and her family expressed their concurrence with the recommended therapeutic strategy.

An assessment upon this admission revealed a performance status (PS) score of 1, indicating the patient was well enough to tolerate chemotherapy. Baseline routine blood and chemistry analyzes revealed no significant abnormalities. Notably, liver function tests were within normal limits (ALB: 42.5 g/L, ALT: 11.4 U/L, AST: 15 U/L, TBIL: 11.3 μmol/L). Contrast-enhanced MRI of the brain revealed punctate enhancing foci in the right insular region and the right cerebellar hemisphere, which are suspicious for metastatic lesions. In addition, pre-existing cerebral ischemic foci were noted in the periventricular white matter and semioval center, which appeared stable compared to the previous examination. The patient displayed no abnormal neurological clinical signs or symptoms. Additionally, potential mechanisms contributing to hyperammonemia, including malnutrition, muscle atrophy, infection, and hepatic or renal dysfunction, were systematically ruled out. Subsequently, systemic chemotherapy (TX) was initiated on 3 January 2023, consisting of liposomal paclitaxel at 150 mg/m^2^ on day 1 and capecitabine at 1,250 mg/m^2^ twice daily from days 1–14 for the initial cycle. The supportive management included the implementation of a low-protein diet aimed at reducing ammonia production, alongside the administration of lactulose to acidify intestinal pH. Additionally, a more intensive therapeutic approach was adopted to mitigate symptoms such as nausea, vomiting, and anorexia.

### 2.4 Second episode of hyperammonemic encephalopathy

#### 2.4.1 Diagnosis and management

On the fourth day of capecitabine therapy, the patient exhibited symptoms including lethargy, decreased appetite, and mild nausea by midday. Subsequently, the patient experienced a loss of consciousness, was unresponsive to stimuli, and presented with rigid extremities, resulting in a Glasgow Coma Scale score of 4 (E1, V1, M2). According to standard clinical classification, a GCS score of 3-8 is indicative of a severe impairment of consciousness. Electrocardiographic assessment showed a heart rate of 82 beats per minute, blood pressure of 126/75 mmHg, and an oxygen saturation of 99%. Physical examination revealed a body temperature of 36.6 °C, pupils approximately 5 mm in diameter with equal size, a sluggish pupillary light reflex, and no abnormalities detected in cardiac and pulmonary auscultation. Additionally, the patient demonstrated neck stiffness, bilateral lower limb rigidity, and bilateral positive Babinski signs. A rapid fingertip blood glucose measurement was 7.5 mmol/L. Arterial blood gas analysis indicated metabolic acidosis with respiratory alkalosis. Hematological analysis revealed a significantly elevated blood ammonia level of 333.60 μmol/L. Liver function tests revealed significantly elevated enzyme levels, with ALT at 180.70 U/L and AST at 227.20 U/L, alongside mild renal dysfunction, indicated by urea at 8.39 mmol/L and creatinine at 84.60 mmol/L (Table 1). Coagulation function tests, troponin T, pro-brain natriuretic peptide, and urinalysis did not reveal any significant abnormalities. A non-contrast computed tomography (CT) scan of the brain showed no apparent abnormalities, and a cranial magnetic resonance imaging (MRI) was not performed. The patient was diagnosed with hyperammonemic encephalopathy and was immediately transferred to the ICU. Presented with marked hepatic impairment, the patient underwent plasmapheresis rather than hemoperfusion for ammonia lowering. Plasmapheresis offers the dual advantage of toxin adsorption and artificial liver support, in contrast to hemoperfusion, which is primarily limited to toxin adsorption. Additionally, intravenous branched-chain amino acids and lactulose enemas were administered to reduce blood ammonia levels (Table 2). Subsequently, the patient developed a fever of 39.0 °C after being transferred to the ICU. Based on a positive sputum culture for *Staphylococcus aureus* and negative blood cultures, intravenous antibiotic therapy was administered.

#### 2.4.2 Outcome

**FIGURE 2:**
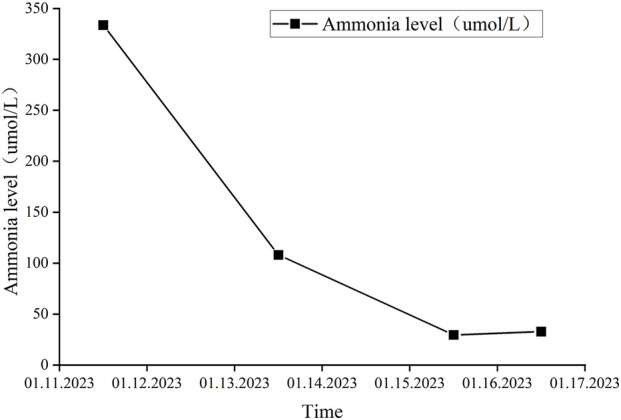
Blood ammonia levels in 2023.

**FIGURE 3:**
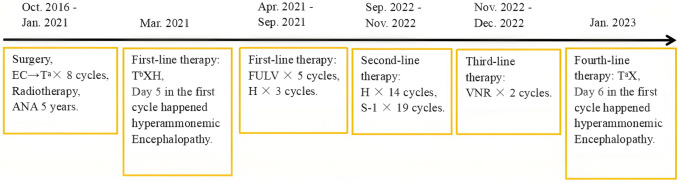
Timeline detailing medical treatments from October 2016 to January 2023. It includes periods of surgery, chemotherapy, radiotherapy, multiple therapy lines, and occurrences of hyperammonemic encephalopathy.

After 7 days of ICU care, the patient regained consciousness, and laboratory parameters, including blood ammonia at 29.5 μmol/L (Figure 2), liver enzymes (ALT 51.90 U/L, AST 46.2 U/L), and lactic acid (1.0 mmol/L), largely normalized. The patient was subsequently transferred to a general ward and discharged upon recovery. Due to the comprehensive communication with the patient’s family prior to treatment initiation, they expressed understanding after the recurrence of hyperammonemic encephalopathy. Despite full recovery and discharge after both episodes, both clinical courses imposed a significant physiological burden on the patient. Following discharge in January 2023, the patient was regularly followed up with close monitoring. She did not receive any further antitumor therapy and succumbed at home 1 year after discharge; the exact cause of death could not be ascertained. The patient’s complete treatment timeline is shown in Figure 3.

## 3 Discussion

### 3.1 Key findings and clinical significance

For the two episodes of hyperammonemic encephalopathy in this patient, hyperammonemia of hepatic origin and other comorbidities capable of altering consciousness were first excluded. Consequently, a drug-induced etiology was suspected. A temporal analysis of the patient’s medication history revealed capecitabine as the drug common to each of the two hyperammonemic episodes. Other agents, including nab-paclitaxel, liposomal paclitaxel, and trastuzumab, were administered prior to only a single event and were thus excluded as primary culprits. Capecitabine was therefore considered the most probable causative drug.

### 3.2 Literature context and pharmacovigilance data

Hyperammonemia, characterized by an abnormal elevation of ammonia levels in the blood, has a multifactorial etiology. While liver failure and other hepatic disorders are the predominant causes of hyperammonemia, emerging evidence underscores the significance of non-hepatic factors. Patients with hyperammonemia frequently require ICU support, and several studies have focused on this specific patient population. Approximately 4% of these patients are reported as having no liver disease (Sakusic et al., 2018). Furthermore, other studies have indicated that up to 18% of hyperammonemic cases are attributable to non-hepatic causes, with 3% linked to drug-related factors (Jacoby et al., 2020). In this context, pharmacovigilance analyzes have further delineated specific drug associations. An analysis of VigiBase identified 71 drugs associated with hyperammonemia, including antitumor, antiepileptic, immunosuppressive, and psychiatric medications. Notably, among anticancer drugs, 5-FU and capecitabine were implicated, whereas taxanes and trastuzumab were not (Balcerac et al., 2022). Similarly, a study examining the Japanese Adverse Drug Event Report Database identified 17 anticancer agents as potential causative factors for hyperammonemia, including 5-FU, capecitabine, and S-1, while excluding taxanes and trastuzumab (Oura et al., 2023).

A major clinical consequence of hyperammonemia is hyperammonemic encephalopathy. Based on current clinical consensus, this condition can be primarily classified into hepatic and non-hepatic types, each with distinct characteristics and prognoses. Hepatic hyperammonemic encephalopathy, the most common form, typically arises from acute liver failure or acute decompensation of chronic liver disease (Clay and Hainline, 2007). Its pathogenesis centers on impaired hepatic urea synthesis and portosystemic shunting. When blood ammonia levels exceed 200 μmol/L, the risk of cerebral edema and intracranial hypertension increases significantly (Clay and Hainline, 2007). Mortality is notably high, ranging from 64% to 86%, particularly among patients with severe hyperammonemia accompanied by acute-on-chronic liver failure (Jacoby et al., 2020). Non-hepatic hyperammonemic encephalopathy encompasses diverse etiologies. Stergachis et al. systematically categorized this entity into four groups (Stergachis et al., 2020). The first is acquired urea cycle dysfunction, frequently triggered by medications (e.g., valproic acid, 5-fluorouracil and its prodrugs), severe malnutrition, high tumor burden, or post-transplant status (Laish and Ben Ari, 2011; Prado et al., 2015; Stergachis et al., 2020). Within this category, 5-fluorouracil-associated encephalopathy represents a rare subtype, with an incidence of approximately 0.6%. It typically manifests rapidly after drug administration as acute encephalopathy with marked hyperammonemia. Sarcopenia is an important predictive marker; however, prognosis is generally favorable upon prompt drug discontinuation and supportive care ( Mitani et al., 2017 ). The second category involves infection with urease-producing organisms, such as Ureaplasma, most commonly observed after solid organ transplantation and capable of causing fulminant hyperammonemia (Stergachis et al., 2020). The third category comprises adult-onset congenital metabolic defects, such as ornithine transcarbamylase deficiency, often precipitated by stressors (Stergachis et al., 2020). The fourth category includes idiopathic or unknown causes, frequently seen in states of intense catabolism (e.g., following bone marrow transplantation) (Stergachis et al., 2020). Overall, non-hepatic hyperammonemic encephalopathy carries a mortality rate of approximately 30%, with established risk factors including malnutrition, gastric bypass surgery, valproic acid use, and renal failure (Sakusic et al., 2018).

### 3.3 Diagnostic reasoning and causality assessment

A thorough clinical evaluation was conducted to investigate the etiology of hyperammonemic encephalopathy, which effectively ruled out potential causes including liver failure, portosystemic shunts, severe malnutrition, post-transplant status, urease-producing organisms infections, following bone marrow transplantation and OTC. Furthermore, no comorbid conditions, including acute cerebrovascular diseases, renal insufficiency, electrolyte imbalances, or disorders of glucose metabolism, were identified that could explain the patient’s altered consciousness. Although after the first episode of hyperammonemia revealed cerebral ischemic foci in the right basal ganglia and semioval center, comparison with pre-chemotherapy cranial MRI demonstrated no significant interval changes. Prior to the second hyperammonemic episode, punctate enhancing lesions were detected in the right insular cortex and right cerebellum, their minute size was deemed insufficient to cause neurological deficits. Similarly, pre-existing cerebral ischemic foci in the periventricular white matter and semioval center appeared stable compared to previous imaging. This was further supported by the absence of any clinical neurological symptoms or signs with the patient before treatment initiation. Additionally, the patient exhibited a bilateral positive Babinski sign during both episodes of acute encephalopathy. This pathological reflex signifies upper motor neuron dysfunction. In the context of acute encephalopathy, its bilateral presence implicates diffuse cerebral involvement, as is characteristic of metabolic, toxic, or global hypoxic-ischemic etiologies, rather than a focal structural lesion like stroke or tumor.

After ruling out the aforementioned causes of hyperammonemia, attention was turned to investigating a potential drug-induced etiology. The patient had previously undergone four cycles of liposome paclitaxel as adjuvant chemotherapy in 2016 and had been receiving trastuzumab for over a year, without any noticeable adverse effects from either treatment. An extensive review of databases such as PubMed and CNKI found no documented cases linking paclitaxel, liposome paclitaxel, or trastuzumab with hyperammonemic encephalopathy. Furthermore, analyzes of the VigiBase and Japanese Adverse Drug Event Report databases revealed no association between taxanes, trastuzumab, and hyperammonemia (Balcerac et al., 2022; Oura et al., 2023). As a result, these drugs were excluded as potential contributors to hyperammonemia in this patient. Although hyperammonemic encephalopathy is not a common adverse drug reaction of capecitabine, it is noteworthy that a literature search of databases including PubMed and CNKI revealed several case reports of capecitabine-induced hyperammonemic encephalopathy published since 2017 (Table 3). Investigations within the VigiBase and the Japanese Adverse Drug Event Report databases indicated a correlation between 5-FU, capecitabine, and hyperammonemia. The patient’s symptoms resolved following the discontinuation of capecitabine and the treatment with ammonia-lowering therapies, such as hemoperfusion or plasma exchange. However, a recurrence of similar symptoms was observed upon readministration of capecitabine. These symptoms could not be explained by other concomitant medications, the patient’s underlying disease state, or other therapeutic interventions. The causal relationship between capecitabine and hyperammonemic encephalopathy was assessed using the Naranjo Adverse Drug Reaction Probability Scale (Naranjo et al., 1981). The Naranjo assessment method primarily summarizes the scores based on Naranjo’s ten detailed rules, dividing the total score results into four levels: “definite”, “probable”, “possible”, “doubtful”. The Naranjo score for capecitabine was 5, corresponding to the level of “probable”, indicating that capecitabine is the most likely cause of hyperammonemic encephalopathy.

**TABLE 3:** Published case reports of capecitabine-induced hyperammonemia (1993–2025).

Author,Year	Female	Age (years)	Primary tumour	drugs	FPs daily dose	Envent in cycle X	Envent on day X	Ammonia level (μmol/L)	Resolution time
Mitani et al. (2017)	Male	62	Colorectal	Capecitabine + Bev	2000 mg/m2,D1-14	11	Day 4	180	After 3 days
	Male	66	Colorectal	Capecitabine + Bev	2000 mg/m2,D1-14	6	Day 15	152	After 7 days
Fang et al. (2018)	Female	57	Colorectal	Capecitabine	1.5 g bid,D1-14	1	Day 5	245	After 5 days
Chen and Hu (2018)	Male	53	Gastric	Capecitabine + DTX + Herceptin	3.5 g qd,D1-14	1	Day 2	210	After 1 days
Chu and Salzman (2019)	Female	67	Gastric	Capecitabine + Carboplatin	1000 mg/m2,D1-14	3	Day 5	158	After 10 days
Di Federico et al. (2020)	Male	65	lung carcinoid	Capecitabine+oxaliplatin	1600 mg/m2,D1-14	1	Day 11	167	After 3 days
Cerrella Cano et al. (2022)	Male	79	Pancreatic	Capecitabine	Not reported	1	Day 2	221	-
Giglio et al. (2025)	Male	60	Colorectal	Capecitabine	1000 mg/m2,D1-14	-	-	178	-

Bev, bevacizumab; DTX, docetaxel Herceptin Trastuzumab, -: Not reported.

### 3.4 Incidence and clinical presentation

5-FU and its analogs infrequently induce hyperammonemia, with reported incidence rates ranging from 0.5% to 5.7% (Nakamura et al., 2020). However, the occurrence of hyperammonemic encephalopathy is even less common, as not all patients with hyperammonemia exhibit encephalopathic symptoms. Current evidence suggests that acute encephalopathy manifests in approximately 70% of patients with hyperammonemia, irrespective of the underlying cause, whether it be liver-related or attributable to non-liver factors. The development of encephalopathy is not correlated with peak blood ammonia levels, although patients experiencing severe hyperammonemia are at an elevated risk for encephalopathy (Sakusic et al., 2018). Instances of postreversible encephalopathy, Wernicke encephalopathy, and hyperammonemic encephalopathy have been documented in association with chemotherapy involving 5-FU and its analogs (Yi et al., 2016). A study reported a 0.6% incidence of acute hyperammonemic encephalopathy following chemotherapy with 5-FU and its analogs. Specifically, the incidence was 0.7% for 5-FU, 0.9% for capecitabine, and 0.1% for S-1 (Mitani et al., 2017). Hyperammonemic encephalopathy often presents with non-specific clinical manifestations, frequently leading to underdiagnosis. Patients with mild hyperammonemia are often asymptomatic but may experience nausea and vomiting, symptoms that are difficult to distinguish from chemotherapy-induced side effects. In contrast, severe hyperammonemia is primarily characterized by neurological manifestations, including altered mental status—the most common presentation—as well as seizure development and cerebral edema (Balcerac et al., 2022). The altered mental status can be further stratified as mild, moderate, or severe suppression based on its severity (Mitani et al., 2017). Importantly, the overall severity of the clinical presentation is closely correlated with a worse prognosis (Sakusic et al., 2018). This patient exhibited neurological abnormalities during both episodes of hyperammonemic encephalopathy. Neuroimaging did not reveal any abnormalities, and aside from elevated blood ammonia levels, no other diagnostic evaluations identified any findings that could explain the altered state of consciousness. This case report indicates that the patient’s altered mental status was attributable to chemotherapy-induced hyperammonemia.

### 3.5 Laboratory and imaging

In cases induced by 5-fluorouracil, the hematological profile is often characterized by marked hyperammonemia (range: 50–940 μmol/L), with neurological manifestations typically emerging at levels >120 μmol/L, and is frequently accompanied by lactic acidosis (7–11 mmol/L, pH 6.99-7.16) (Chahin et al., 2020; Asano et al., 2024). Renal impairment (eGFR <60 mL/min/1.73 m^2^) is a key predisposing factor, whereas liver function is often normal (Yamasaki et al., 2023; Jin et al., 2025). CT of the brain is usually unremarkable, while contrast MRI of the brain may reveal PRES-like changes, including T2/FLAIR hyperintensities in the bilateral cerebellar peduncles, posterior limbs of the internal capsules, and parieto-occipital lobes, with restricted diffusion on DWI suggesting metabolic injury (Kim et al., 2023; Furukawa et al., 2024). This syndrome remains a diagnosis of exclusion, as no definitive diagnostic criteria have been established. On both episodes of this patient, the peak plasma ammonia level exceeded 120 μmol/L, accompanied by varying degrees of lactic acidosis. Although renal function (eGFR >60 mL/min/1.73 m^2^) and liver biochemistry were normal prior to each episode, laboratory investigations during the acute phase revealed transient transaminitis. This hepatic involvement was more prominent in the second episode, a feature potentially associated with the administration of liposomal paclitaxel. Non-contrast CT of the brain performed after both episodes showed no significant abnormalities. A non-contrast MRI of the brain obtained after the first episode demonstrated no PRES-like changes, which may reflect the limited sensitivity of non-contrast MRI in this context; regrettably, no MRI of the brain was performed following the second episode. This clinical course highlights the importance of performing a comprehensive diagnostic evaluation to improve the diagnostic accuracy and inform management strategies in similar complex cases.

### 3.6 Pathophysiological mechanisms

The pathophysiology of fluorouracil-induced hyperammonemic encephalopathy remains uncertain, likely involving multiple contributing factors. The most frequently proposed mechanism is a deficiency in dihydropyrimidine dehydrogenase (DPD), an enzyme responsible for the degradation of approximately 80% of 5-FU, with about 10% being excreted unchanged (Jin et al., 2025). Patients with DPD deficiency are at a significantly increased risk of severe toxicities, including diarrhea, neutropenia, and mucositis, with a subset of these patients also experiencing neurotoxicity and, in rare cases, coma (Morel et al., 2006). In the context of DPD deficiency, the impaired metabolism of 5-FU does not fully account for the occurrence of hyperammonemia. Given the hydrophilic nature of 5-FU, it is unable to readily cross the blood-brain barrier, suggesting that the accumulation of 5-FU alone does not explain the onset of acute encephalopathy (Alzahrani et al., 2023). Nonetheless, some researchers have reported that excessive ingestion of 5-FU may allow it to penetrate the cerebrospinal fluid, potentially inducing neurotoxic effects and contributing to the development of Wernicke’s encephalopathy (Yi et al., 2016). It should be noted that DPD level assessment is not routinely conducted at our institution, and the DPD status of the patient was not determined. The patient developed grade II-III thrombocytopenia following both hyperammonemic episodes, these adverse events more plausibly linked to the prior administration of nab-paclitaxel and liposome paclitaxel. Notably, the clinical presentation was not accompanied by significant mucositis or grade III-IV neutropenia. The absence of these characteristic toxicities makes the likelihood of an underlying DPD deficiency comparatively low.

Another possible mechanism for this phenomenon involves 5-FU metabolites. The enzyme DPD metabolizes 5-FU into ammonia and fluoro-β-alanine, which is subsequently converted into fluoroacetate. Fluoroacetate inhibits the tricarboxylic acid (TCA) cycle by interfering with citrate, thereby inhibiting the production of adenosine triphosphate (ATP) and oxaloacetate, both of which are essential for the urea cycle. Overproduction of fluoroacetate disrupts the TCA cycle, consequently affecting the urea cycle. This dysfunction impairs normal ammonia metabolism, leading to its accumulation in the body and resulting in hyperammonemia (Boilève et al., 2019). We suggest an alternative hypothesis is that the accumulation of 5-FU in the body may affect metabolism through various mechanisms, or underlying conditions in these patients may contribute to abnormal neurological changes. Further investigation is warranted.

### 3.7 Differential toxicity: S-1 vs. capecitabine

**FIGURE 4:**
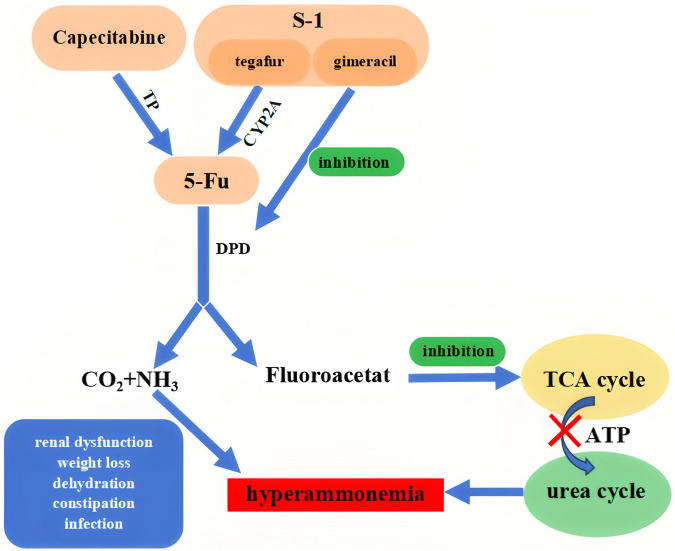
Mechanisms by which capecitabine and S-1 cause hyperammonemia. TP, Thymidine phosphorylase; CYP2A6, Cytochrome P450 2A6; DPD, Dihydropyrimidine dehydrogenase.

Patients exhibit better tolerance to S-1 compared to capecitabine, likely attributable to differences in drug design. S-1 is a formulation comprising tegafur, gimeracil (CDHP), and oteracil potassium. Tegafur acts as a prodrug of 5-FU, undergoing continuous conversion to 5-FU primarily mediated by cytochrome P450 2A6 (CYP2A6) following oral administration, thereby exerting its anticancer effects (Wang et al., 2011). CDHP functions as an inhibitor of DPD, thereby reducing the degradation of 5-FU. This mechanism, in comparison to tegafur alone, enhances the plasma levels of 5-FU and maintains its effective concentration in both plasma and tumor tissues (Saif et al., 2009). Capecitabine, a fluorouracil carbamate antineoplastic agent, undergoes metabolic conversion to 5-FU within tumor cells following oral administration. This transformation is facilitated by the enzymatic activities of carboxylesterase and cytidine deaminase in both hepatic and tumor tissues, with thymidine phosphorylase (TP) catalyzing the final step, thereby achieving a targeted antineoplastic effect (Alzahrani et al., 2023). Compared to S-1, capecitabine is more rapidly converted to 5-FU and lacks mechanisms to impede the degradation of 5-FU. The extensive breakdown of 5-FU may lead to the production of significant amounts of fluoroacetate, which has the potential to disrupt the urea cycle. This disruption can hinder ammonia metabolism, resulting in its accumulation in the body and consequently causing hyperammonemia (Figure 4).

### 3.8 Contributing risk factors

5-FU monotherapy has generally not been associated with hyperammonemia. However, interactions between renal dysfunction and 5-FU can precipitate this condition, and the use of fluoropyrimidine may elevate the risk of hyperammonemia in patients with chronic kidney disease (Oura et al., 2023). Factors such as weight loss, dehydration, constipation, and bacterial infections can enhance the activity of bacterial urease and amino acid oxidase. Urinary tract infections caused by urease-producing bacteria have been linked to hyperammonemia, with these mechanisms functioning independently of the presence of 5-FU (Chahin et al., 2020). In this patient, a thorough evaluation during the first hyperammonemic episode excluded common metabolic precipitants (e.g., infection, fasting, surgery, or trauma) and the contribution of home medications. Although the second episode was complicated by a concurrent pulmonary infection, *S. aureus* is not a urease-producing organism, and no other clear triggers for metabolic imbalance or suspect drug exposures were identified. Notably, during both episodes, the patient developed varying degrees of concurrent hepatic and renal dysfunction, which likely compromised ammonia clearance. Furthermore, chemotherapy-related adverse effects-including weight loss, dehydration, and tumor lysis-collectively contributed to increased ammonia production. The interplay of these factors ultimately led to the significant hyperammonemia observed.

### 3.9 Proposed treatment

Immediate cessation of fluoropyrimidine administration constitutes the cornerstone of management. First-line therapy comprises aggressive intravenous hydration to reverse dehydration—a precipitating factor in 73% of cases—and branched-chain amino acids infusion, employed as the primary ammonia-lowering strategy. For mild-to-moderate encephalopathy, second-line pharmacologic interventions include oral/enema lactulose to acidify the colonic lumen, ornithine aspartate to activate the urea cycle, and oral rifaximin/neomycin to suppress intestinal ammonia production. Continuous hemodiafiltration (CHDF) is reserved for severe presentations complicated by lactic acidosis or hemodynamic instability, achieving 79.5% clearance of 5-FU. With prompt intervention, median recovery time is 2 days (range <1-7), with ammonia normalization within 48 h and no long-term neurologic sequelae. Prophylactic strategies should prioritize identifying predisposing factors (sarcopenia present in 91% of cases, renal dysfunction, infection) and monitoring serum ammonia 24–72 h post-chemotherapy in high-risk individuals (Mitani et al., 2017).

### 3.10 Clinical implications and take-home messages

This case underscores capecitabine as a rare but critical cause of hyperammonemic encephalopathy in oncology patients. It highlights a paramount clinical lesson: in any patient receiving fluoropyrimidines who presents with acute mental status changes, drug-induced hyperammonemia must be considered an emergent differential diagnosis, even in the presence of normal baseline liver and renal function. A systematic diagnostic approach is essential-first ruling out common causes like liver failure and severe infection, then meticulously reviewing the medication history for potential culprits. Critically, management must not be delayed awaiting confirmatory tests; immediate discontinuation of the suspected drug and prompt initiation of ammonia-lowering therapies are the cornerstones of treatment and are vital for preventing severe neurological sequelae. Heightened clinician awareness of this adverse event can lead to earlier intervention and improved patient outcomes.

## 4 Conclusion

Hyperammonemic encephalopathy is a rare adverse effect associated with 5-FU and its analogs, often overlooked or misdiagnosed in routine clinical practice. When other diagnostic investigations fail to identify the etiology of unexplained neurological symptoms in patients undergoing treatment with these agents, it is imperative to consider the possibility of drug-induced hyperammonemic encephalopathy. Discontinuation of the offending drug is crucial, and symptomatic management should include prompt fluid resuscitation, administration of branched-chain amino acids, and lactulose enemas. Hemodialysis may be employed to reduce elevated blood ammonia levels. Monitoring DPD enzyme activity, DPYD gene mutations, and TCA cycle substrates can provide valuable insights for further therapeutic strategies when feasible. Furthermore, heightened vigilance for plasma ammonia monitoring is warranted in high-risk populations, including patients with renal failure, malnutrition, valproate use, or ornithine transcarbamylase (OTC) deficiency. Although hyperammonemia itself is not an independent predictor of mortality, it is associated with poor overall prognosis, necessitating early recognition and intervention. Future studies should focus on elucidating the mechanisms and developing targeted therapeutic strategies for non-hepatic hyperammonemia, including cases associated with 5-fluorouracil and its analogs. This article aims to enhance awareness regarding the diagnosis and management of this condition, prevent its recurrence, and improve patient outcomes, thereby advancing clinical practice.

## Data Availability

The original contributions presented in the study are included in the article/supplementary material, further inquiries can be directed to the corresponding author.
